# The Relationship between Serum Bilirubin and Elevated Fibrotic Indices among HBV Carriers: A Cross-Sectional Study of a Chinese Population

**DOI:** 10.3390/ijms17122057

**Published:** 2016-12-09

**Authors:** Min Du, Shanshan Zhang, Lin Xiao, Yanyan Xu, Peiyi Liu, Yuhan Tang, Sheng Wei, Mingyou Xing, Xiaoping Miao, Ping Yao

**Affiliations:** 1 Department of Nutrition and Food Hygiene, Hubei Key Laboratory of Food Nutrition and Safety and the Ministry of Education (MOE) Key Lab of Environment and Health, School of Public Health, Tongji Medical College, Huazhong University of Science and Technology, 13 Hangkong Rd., Wuhan 430030, China; treacy29@163.com (M.D.); 13035130764@163.com (S.Z.); xiaolin0210@hust.edu.cn (L.X.); yyx@hust.edu.cn (Y.X.); liupeiyi85@126.com (P.L.); tyh043@126.com (Y.T.); 2 Department of Epidemiology and Biostatistics and the Ministry of Education (MOE) Key Lab of Environment and Health, School of Public Health, Tongji Medical College, Huazhong University of Science and Technology, 13 Hangkong Rd., Wuhan 430030, China; ws2008cn@gmail.com; 3 Department of Infectious Disease, Tongji Hospital of Tongji Medical College, Huazhong University of Science and Technology, 13 Hangkong Rd., Wuhan 430030, China; xingmingyou@126.com

**Keywords:** bilirubin, liver fibrosis, hepatitis B surface antigen (HBsAg)-positive, cross-sectional study

## Abstract

The study probed the association between bilirubin and hepatitis B virus (HBV) infection and progression. A cross-sectional analysis of 28,500 middle aged and elderly Chinese participants was performed to analyze the differences of bilirubin in terms of hepatitis B surface antigen (HBsAg) positive or negative and the correlation between bilirubin and severity of hepatic fibrosis estimated by non-invasive indices. Bilirubin was significantly higher in the HBsAg (+) group than the HBsAg (−) group. Higher bilirubin levels were consistently associated with elevated liver fibrosis indices among HBsAg carriers. Compared with quartile 1 of total bilirubin (TBil), the multivariable-adjusted ORs (95% CIs) for elevated fibrosis indices of quartile 4 were 2.24 (95% CIs, 1.57–3.21) estimated by fibrosis 4 score (FIB-4) and 2.22 (95% CIs, 1.60–3.08) estimated by aspartate transaminase to platelet ratio index (APRI). In addition, direct bilirubin (DBil) had a stronger association with elevated liver fibrosis indices than did indirect bilirubin (IBil). Furthermore, the relationship between DBil and elevated fibrosis indices was more robust among participants who were female, overweight or had central fat distribution. These findings suggested that bilirubin levels, especially DBil, were independently associated with an increased risk of increased fibrosis indices.

## 1. Introduction

Although the hepatitis B vaccine is available, more than 350 million people are chronically infected with hepatitis B virus (HBV) [1], and about 30% of the world’s population shows serological evidence of current or past infection [2]. HBV infection is a major threat to public health, especially in China [3]. It has been estimated that more than 80% of liver cancer worldwide is attributable to hepatitis B or C virus infections [4]. Patients with HBV infection have a high risk of progressive liver fibrosis which can lead to cirrhosis and hepatocellular carcinoma (HCC). In addition, inflammatory milieu caused by chronic viral infections might influence hepatic glucose sensitivity and increase insulin resistance [5], which could be determined from the findings that diabetes and prediabetes were prevalent among HBV-infected patients [6]. HBV infection is the tenth leading cause of death worldwide, with about 786,000 related deaths every year [7]. Therefore, HBV infection causes high mortality and creates a social burden.

Bilirubin, a primary end product of heme catabolism, processes cytoprotective properties because of the antioxidant nature of the bile pigment. In 1995, it was first suggested by Breimer that bilirubin might be implicated in the protection of specific kinds of diseases resulting from oxidative damage [8]. Then, as observed by related reports, bilirubin appeared to have the innate capacity to resist oxidative damage [9,10,11]. Meanwhile, all patterns of serum bilirubin, including total bilirubin (TBil), direct bilirubin (DBil) and indirect bilirubin (IBil), display protective properties in cardiovascular diseases [12]. Several studies clarified that the robust anti-oxidative properties of bilirubin could largely explain its protective effects [13,14,15], and the findings that subjects with higher serum bilirubin had elevated total antioxidant status also confirmed its anti-oxidative property [16]. 

On the other hand, bilirubin has previously been proven to be a marker of liver injury and is incorporated in several prognostic scoring models, such as the Child–Pugh (CP) score and the model of end-stage liver disease (MELD) [17]. In recent years, relevant studies focused on the effect of bilirubin on several hepatic disorders. Recent study suggested that DBil independently reduced non-alcoholic fatty liver disease (NAFLD) risk [18]. Patients who were with liver biopsy-proved non-alcoholic steatohepatitis (NASH) had significantly lower bilirubin levels compared with those without NASH, and there was also an inverse association between bilirubin levels and histological features including fibrosis [19,20]. Serum IBil levels were negatively correlated with the progression of liver fibrosis in chronic hepatitis C (CHC) patients [21]. However, the concentrations of serum bilirubin increased along with the increased severity of fibrosis among CHC patients [22]. High levels of bilirubin or combined prognostic index including bilirubin were able to predict short-term mortality in the patients with acute-on-chronic liver failure [23,24]. Meantime, abnormal bilirubin values were even more strongly associated with poor clinical outcome at baseline and up to five years follow-up in the patients with primary billiary cirrhosis [25]. 

Related studies have illustrated the associations between bilirubin and liver disease. However, the study which is performed on participants with HBV infection is lacking. Secondly, the most of these studies did not investigate the associations between all subtypes of bilirubin and liver disease. Finally, the study that assesses the relationship between bilirubin and HBV-related fibrosis based on large sample sizes might be needed.

Considering the apparently bewildering complexity of bilirubin’s function in different milieus and the high prevalence of HBV infection, the underlying association between bilirubin and liver fibrosis with HBV infection needs to be warranted. The task for this study is to untangle the intrinsic relationship between bilirubin and the different indices reflecting liver function in health check-ups with HBV infection. 

## 2. Results

### 2.1. Characteristics of Participants

A total of 28,500 participants (26,549 with HBsAg negative, 1951 HBsAg positive) were included. Demographics and laboratory data of the subjects are listed in Table 1. Firstly, individuals with HBsAg positive were younger than control subjects. HBsAg seropositive subjects had a higher prevalence of current smoking and drinking, which might be explained by the fact that the individuals with HBV infection had more males compared to HBsAg negative subjects. Then, HBsAg seropositive individuals had a lower prevalence of traditional cardiovascular risk factors like hypertension, diabetes, coronary heart disease (CHD) and fatty liver. Next, the mean levels of platelet count, total cholesterol (TC), triglycerides (TG) and low-density lipoprotein cholesterol (LDL-C) were significantly lower in the HBsAg (+) group. As expected, subjects who were HBsAg-positive had higher levels of liver injury markers (aspartate transaminase (AST) and alanine transaminase (ALT)) compared with HBsAg-negative subjects. It was notable that mean levels of TBil, IBil and DBil were significantly higher in the HBsAg (+) group than the control group.

### 2.2. Associations between Serum Bilirubin and Demographic, Biochemical Parameters, Non-Invasive Liver Fibrosis Indices among HBsAg (+) Participants

Among the carriers of HBV, the associations between serum bilirubin and demographic, biochemical parameters, non-invasive liver fibrosis indices are presented in Table 2. TBil showed significant associations with age, waist-to-hip ratios (WHR), AST, hemoglobin, platelets count, TG, TC, LDL-C, aspartate transaminase to platelet ratio index (APRI) and Fibrosis 4 score (FIB-4). Surprisingly, DBil exhibited significant associations with more parameters than IBil, possibly reflecting the differences of the two forms of bilirubin. In both DBil and IDil, we observed positive associations with age, WHR, hemoglobin, APRI and FIB-4, and inverse association with platelets count and TC. The positive correlations between DBil and the liver injury makers (AST) were statistically significant, but the significant associations between IBil and certain markers of liver injury (AST and ALT) were not shown.

### 2.3. Serum TBil Levels in Relation to Fibrotic Indices among HBsAg (+) Participants

Table 3 shows that there are sequentially higher odds of elevated liver fibrosis indices with ascending quartiles of TBil in multivariate models. After adjusting for age, sex, body mass index (BMI), WHR, smoking, drinking, education, marriage status and physical activity, TBil level increase was still significantly linked to the risks of elevated APRI and FIB-4. The positive relationship was decreased by additional adjustment for medical history but still statistically significant. The corresponding odds ratios (ORs) (95% confidence intervals (CIs)) for risks of elevated APRI comparing the upper 3 TBil quartiles with the lowest TBil quartile were 1.26 (0.90, 1.78), 1.78 (1.28, 2.47), 2.22 (1.60, 3.08). As for elevated FIB-4, corresponding ORs (95% CIs) comparing the upper 3 TBil quartiles with the lowest TBil quartile were 1.57 (1.14, 2.17), 1.64 (1.18, 2.28), 2.24 (1.57, 3.21). 

### 2.4. Associations between Different Forms of Bilirubin (IBil and DBil) and Fibrosis Scores among HBsAg (+) Participants

We further analyzed the associations between different forms of bilirubin (IBil and DBil) and fibrosis score among HBsAg (+) participants. The positive associations were found between IBil with the elevated APRI or FIB-4 (Table S1). Corresponding ORs (95% CIs) for elevated APRI comparing the upper 3 IBil quartiles with the lowest IBil quartile were 1.30 (0.93, 1.82), 1.52 (1.10, 2.11), 2.16 (1.57, 2.98) after multi-adjustment. As for elevated FIB-4, the corresponding ORs (95% CIs) comparing the upper 3 IBil quartiles with the lowest IBil quartile were 1.24 (0.89, 1.72), 1.41 (1.01, 1.96), 1.75 (1.24, 2.49). 

A similar tendency was exhibited between DBil and the two fibrosis indices (Table S2). Corresponding ORs (95% CIs) for elevated APRI comparing the highest DBil quartile with the lowest IBil quartile was 2.64 (1.89, 3.70) after full adjustment. As for elevated FIB-4, the corresponding ORs (95% CIs) comparing the highest DBil quartile with the lowest DBil quartile was 3.07 (2.10, 4.50). The trend between bilirubin and elevated fibrotic indices was statistically significant for IBil (*P*_FIB-4_ < 0.001 and *P*_APRI_ < 0.001) and DBil (*P*_FIB-4_ < 0.001 and *P*_APRI_ < 0.001). Moreover, the fully adjusted ORs (95% CIs) for DBil (Q4 vs. Q1: ORs for FIB-4, 3.07 (95% CIs: 2.10, 4.50); ORs for APRI, 2.64 (95% CIs: 1.89, 3.70)) were larger than those for IBil (Q4 vs. Q1: ORs for FIB-4, 1.75 (95% CIs: 1.24, 2.49); ORs for APRI, 2.16 (95% CIs: 1.57, 2.98)).

### 2.5. Comparisons of TBil, IBil and DBil in HBsAg(+) Participants

The areas under the receiver operating characteristic curve (AUROC) that predicts elevated APRI and FIB-4 for each form of bilirubin is presented in Table 4. The AUROC values of TBil, IBil and DBil were 0.61 (0.58, 0.64), 0.59 (0.56, 0.62) and 0.63 (0.60, 0.66) for elevated APRI, respectively. As for FIB-4, the corresponding AUC values were 0.61 (0.58, 0.64), 0.57 (0.54, 0.60) and 0.65 (0.62, 0.68), respectively (Table 4). The DBil had higher AUROCs than TBil and IBil.

### 2.6. Serum DBil Levels in Relation to Fibrotic Features in Subgroups among HBsAg(+) Participants

**Figure 1:**
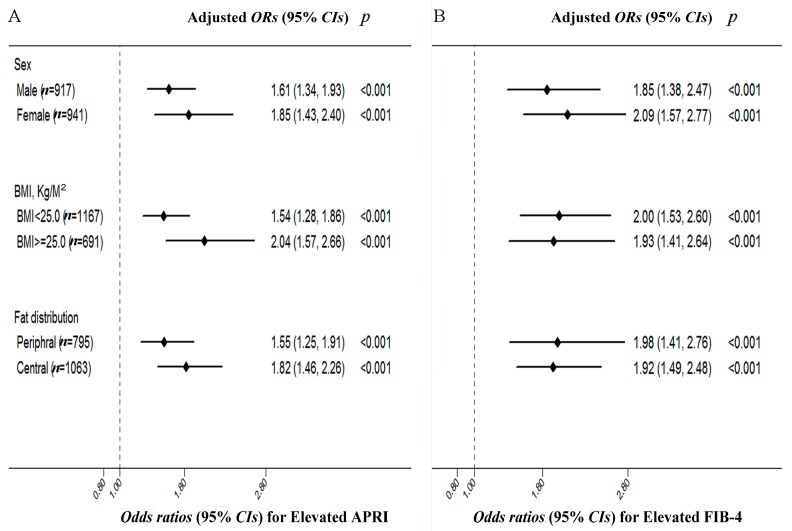
The risks of elevated APRI (**A**) and FIB-4 (**B**) with each 1 SD increase in serum DBil among HBsAg(+) participants. The risks of elevated APRI (**A**) and FIB-4 (**B**) with each 1 SD increase in serum DBil concentrations according to subgroups of sex (male vs. female), overweight (BMI < 25.0 vs. ≥25.0 kg/m^2^), fat distribution (WHR ≥ 0.92 for male and WHR ≥ 0.81 for female vs. WHR < 0.92 for male and WHR < 0.81 for female). Adjusted for age (continuous), sex (male, female), BMI (continuous), WHR (continuous), smoking (never smoking, quit smoking, current smoking), drinking (never drinking, quit drinking, current drinking), education (≤6/7–9/10–12/≥13), marriage status (yes/no), physical activity (yes/no) and medical history (yes/no for hypertension, CHD, diabetes, fatty liver). APRI, aspartate transaminase to platelet ratio index; FIB-4, Fibrosis 4 score; SD, standard deviation; WHR, waist-to-hip ratios; ORs, odds ratios; CIs, confidence intervals.

To better understand the effect of DBil levels on the liver fibrotic progression across sex, BMI and WHR, we inquired into the relationship between DBil and fibrosis indices in subgroups. Figure 1 shows the risks of elevated fibrosis indices with each standard deviation (SD) increase in DBil in the different sex, overweight or not, central and peripheral fat distribution subgroups. Positive associations between DBil and elevated FIB-4 or APRI were consistent in different subgroups after full adjustment. Subjects who were female were inclined to have a higher risk of elevated fibrosis indices. In addition, after full adjustment, subjects who were overweight or had central fat distribution were inclined to have a higher risk of elevated fibrosis index reflected by APRI than those without these metabolic disorder features. The ORs (95% CIs) for elevated APRI per 1 SD increase of DBil across overweight (yes or no) were 2.04 (1.57–2.66) vs. 1.54 (1.28–1.86). At the same time, the ORs (95% CIs) for elevated APRI per 1 SD increase of DBil between central and peripheral fat distribution were 1.82 (1.46–2.26) vs. 1.55 (1.25–1.91).

## 3. Discussion

In this study, we observed the significant differences in serum bilirubin between individuals with or without HBV infection. Moreover, higher bilirubin levels might indicate more advanced liver fibrosis in a large group of retired workers with serum evidence of HBV infection. In addition, such associations were statistically significant when adjusted for multiple parameters, especially in female, overweight or central fat distribution individuals.

Our observations could probably best explain the relationship between bilirubin and validated non-invasive fibrosis indices among HBV carriers. For a start, a lower prevalence of traditional cardiovascular risk factors like hypertension, diabetes, CHD and fatty liver was observed in the report, which was different from the findings that diabetes and prediabetes were prevalent among HBV-infected patients [6]. One possible reason for this would be the distinct characteristics of the two study groups. Following the phenomenon that the bilirubin levels among HBV carriers were higher than the others, we sought to investigate the relationship between bilirubin and the liver fibrosis among individuals with HBV infection. Unlike the meaningful findings that higher bilirubin concentrations were associated with reduced risk of cardiovascular disease, respiratory illness and mortality in epidemiological studies [13,15], the protective effect of bilirubin was not meaningful in our study. Similar tendency was observed in patients with hepatitis C virus (HCV) associated fibrosis [22], but the concentrations of bilirubin in this study were far beyond the”normal range”. Conversely, the principal results in our study were different from another HCV-related fibrosis study [21] in which the inverse relationship between bilirubin and liver fibrosis was found. The possible reasons for the inconsistent results of the researches above might be the sample size and diverse study design. The results presented indicated that bilirubin might act as an independent risk factor for significant liver fibrosis. Is this biologically plausible? The process of liver fibrosis is the excessive accumulation of extracellular matrix proteins including collagen. Most types of chronic liver disease without clinical symptoms have developed into liver fibrosis which could result in cirrhosis, liver failure, and other severe complications. In advanced cirrhosis, glucuronyl conjugation of bilirubin and biliary excretion of DBil are markedly impaired and jaundice appears [26]. Therefore, the concentration of bilirubin in serum may be a good prognostic marker for patients with decompensated liver cirrhosis. Hepatic fibrosis, as the onset of liver cirrhosis, might disturb the bilirubin’s normal production and excretion in the liver. Although it is difficult to determine the exact mechanisms behind the relationship between bilirubin and fibrotic progression because of complexity of the disease, several hints could be identified. Firstly, related studies proposed that bilirubin was able to induce cytotoxic effects [27,28,29,30], unbalance the redox homeostasis [31], and finally affect the mitochondrial integrity and induce apoptosis [32]. Second, activated retinoid-storing hepatic stellate cells might contribute more to the elevation of DBil levels in blood [33], and the medicine associated with reversion and prevention of cirrhosis could also reduce the levels of serum bilirubin [34]. Lastly, slightly elevated bilirubin could induce a stress response to the endoplasmic reticulum, resulting in a decreased proliferative and metabolic activity of hepatocytes [35].

Three important findings were achieved about evaluating serum bilirubin in individuals with HBV infection. First, our results showed the positive associations between all forms of bilirubin and severity of liver disease. DBil was more correlative with the indices of liver fibrosis. Also, DBil had higher AUROCs than TBil and IBil. To be noticed, there were slight differences between DBil and IBil. IBil had more potent anti-oxidant capacity than the DBil [36] might explain the weaker association between IBil and the risk of elevated fibrosis indices. Second, our results suggested that females should pay more attention to an increase of DBil level. Related study suggested a protective effect of estrogens on fibrogenesis via the inhibition of stellate cell proliferation [37]. The individuals in the current study were featured with old age and estrogens levels were substantially decreased. The findings suggested that elderly females should pay more attention to the increase in DBil. In addition, overweight subjects and those with central fat distribution should also be careful of an increase in DBil level. The intricate interplay between HBV infection and metabolic factors might be involved in the positive relationship between bilirubin and fibrosis [38]. The data in the present study confirmed this idea which the participants with higher DBil levels had higher risks of elevated fibrosis scores among subjects with central fat distribution or overweight compared to those without such metabolic disorders. Lifestyle advice should be offered to all HBsAg carriers due to its easy implementation with little risk of side-effects or cost.

The data displayed here can be explained only in the context of the study design. Firstly, due to the limitations of the cross-sectional study, the potentially important function of bilirubin needed to be further investigated. Second, the severity of liver fibrosis was assessed only by noninvasive indices. Although liver pathology is the gold standard, patients’ discomfort and expense should be considered also. Noninvasive methods had overcome the limitations of liver biopsy and were also used as prognostic indices for subjects with hepatitis B-associated HCC [39]. Furthermore, the accuracy of FIB-4 and APRI were 78% and 76% [40], suggesting they were suitable for regular monitoring of disease progression [41,42]. Thus, using APRI and FIB-4 to assess liver fibrosis was acceptable in the circumstances of the study. Finally, we failed to acquire information on concentrations of virus titer that were potentially linked to the pathophysiology of CHB. The proportion of HBeAg(+) was 1.8%, and relatively small in the study.

In summary, our study demonstrates a robust association between bilirubin levels and higher surrogate indices of liver fibrosis among participants with HBV infection. This suggests that bilirubin levels, especially DBil, were independently associated with an increased risk of increased fibrosis indices.

## 4. Methods

### 4.1. Study Population

We conducted a cross-sectional study using the data from the Dongfeng-Tongji cohort study of retired worker as described previously [43]. In 2013, a total of 38,295 individuals were subjected to physical examination, laboratory tests and accomplished semi-structured questionnaires which included socio-demographic information and other concerned information during face-to-face interviews. Among these subjects, 28,500 underwent laboratory testing for HBV infection. HBV infection was defined as the presence of hepatitis B surface antigen (HBsAg) in peripheral blood. Participants were divided into 2 study groups: (a) participants with HBsAg; (b) controls without HBsAg.

### 4.2. Ethics Statement

Participants were enrolled after obtaining their written informed consent to the study protocol that was approved by the Medical Ethics Committee of the School of Public Health, Tongji Medical College, Huazhong University of Science and Technology and Dongfeng General Hospital, DMC (approval No. 03, 1 August 2008).

### 4.3. Measurements

Participants who underwent an overnight fast were given a physical examination at Dongfeng Central Hospital with trained physicians, nurses and technicians. Body mass index (BMI) was calculated as weight in kilograms/(height in m)^2^. A reasonable estimation of fat distribution might be made using waist-to-hip ratios (WHR). Subjects were separated into those with central fat distribution (WHR ≥ 0.81 for women and ≥ 0.92 for men) and those with peripheral fat distribution (WHR < 0.81 for women and <0.92 for men), as described in related study [44]. After an overnight fast, all blood specimens were collected to test blood lipids, fasting glucose, hepatic function and renal function at the hospital’s laboratory by the ARCHITECT Ci8200 automatic analyzer (Abbott, Chicago, IL, USA) with corresponding reagent kits. The laboratory also provided a complete blood count and urine routine test. Commercially available enzyme immunoassays were used to determine serum HBsAg, hepatitis B e antigen (HBeAg), antibodies to hepatitis B surface antigen, hepatitis B e antigen and hepatitis B core antigen at the same laboratory using a fully automatic immunoanalyzer, Uranus AE 120 (AIKANG, Shenzhen, China). In addition, abdominal B-type ultrasound was inspected using Aplio XG (TOSHIBA, Tokyo, Japan), by experienced radiologists.

A history of regular smoking was defined as having smoked at least one cigarette per day for more than six months. Smokers who met the definition of regular smoking were divided into current smokers and former smokers according to whether these subjects quitted smoking at the time of the interview. The participants never smoking were defined as non-smokers. Likewise, drinking status was classified into three groups: never drinking, quit drinking, current drinking. CHD, hypertension and diabetes were self-reported chronic diseases. The diagnosis of these conditions was used in accordance with well-accepted international standards [45]. The existence of fatty liver was based on the information of abdominal B-type ultrasound.

### 4.4. Indices of Liver Fibrosis

We calculated two validated non-invasive indices for liver fibrosis, including Fibrosis 4 score (FIB-4), aspartate transaminase to platelet ratio index (APRI). All of them were obtained according to the published formula, as previously described [46,47,48].

FIB−4=Age (years)×AST (U/L)Platelet count (109L)×[ALT (U/L)]1/2

APRI=AST (/ULN#)Platelet count (109L)×100

(# where ULN = upper limit of normal for that laboratory, the upper limit of normal for both AST and ALT was 40 U/L).

Elevated FIB-4 and APRI were defined as FIB-4 ≥ 1.45 [40], APRI ≥ 0.5 [40,49], in both male and female.

### 4.5. Statistical Analyses

All statistical analyses displayed in tables were performed using SAS 9.4 software (SAS Institute Inc., Cary, NC, USA). A two-sided *p* value (<0.05) was considered to be of statistical significance. Continuous variables were represented as mean (SD) and further compared using independent *t* tests. Categorical variables were represented as percentages. A chi-square test was used to determine the distribution of categorical variables among various groups. Spearman rank correlation was performed to test the relationship between continuous variables. To identify whether bilirubin levels were associated with the possibility of increased fibrosis-related indices, multivariate logistic regression analysis was conducted to correct important factors. The Cochran–Armitage trend test was used to investigate the trend among binomial proportion of disease progression.

## Figures and Tables

**Table 1:** The characteristics of 28,500 participants.

Characteristics	HBsAg (−)	HBsAg (+)	*p*-Value
*N* (participants)	26,549	1951	
Age (years) *	64.4 (8.5)	62.8 (7.8)	<0.001
Age (years) ^#^	65 (58, 70)	63 (57, 67)	<0.001
Female (%) ^^^	56.6	50.6	<0.001
Education (≤6/7–9/10–12/≥13 %) ^^^	22.6/37.1/28.7/11.6	24.7/37.8/28.1/9.4	0.010
Marriage status (yes %) ^^^	87.7	90.4	0.001
Smoking (never/quit/current %) ^^^	72.4/12.0/15.6	69.0/12.7/18.3	0.003
Drinking (never/quit/current %) ^^^	70.0/5.9/24.1	67.0/7.2/25.8	0.009
Physical activity (yes %) ^^^	89.1	88.5	0.383
BMI (kg/m^2^) *	24.2 (3.3)	24.0 (3.2)	0.009
WHR *	0.88 (0.06)	0.88 (0.06)	0.569
Medical history			
Hypertension (yes %) ^^^	40.4	37.3	0.007
Diabetes (yes %) ^^^	14.1	10.7	<0.001
CHD (yes %) ^^^	16.0	11.4	<0.001
Fatty liver (yes %) ^^^	37.2	31.9	<0.001
Laboratory tests			
ALT (U/L) *	21.2 (16.3)	26.7 (28.6)	<0.001
AST (U/L) *	23.8 (17.5)	28.7 (20.2)	<0.001
Total bilirubin (mg/dL) *	0.86 (0.36)	0.97 (0.42)	<0.001
Indirect bilirubin (mg/dL) *	0.60 (0.26)	0.66 (0.30)	<0.001
Direct bilirubin (mg/dL) *	0.26 (0.14)	0.31 (0.19)	<0.001
Hemoglobin (g/L) *	136.5 (13.8)	138.4 (14.6)	<0.001
Platelets count (10^9^/L) *	198.1 (52.1)	178.6 (53.5)	<0.001
TG (mg/dL) *	135.04 (96.53)	114.07 (72.02)	<0.001
TC (mg/dL) *	187.96 (38.02)	175.88 (34.31)	<0.001
LDL-C (mg/dL) *	109.63 (31.96)	102.53 (28.88)	<0.001

Data are expressed as mean (standard deviation) *, median (interquartile range) ^#^ or % ^^^. HBsAg, hepatitis B surface antigen; BMI, body mass index; WHR, waist-to-hip ratios; CHD, coronary heart disease; ALT, alanine transaminase; AST, aspartate transaminase; TG, triglycerides; TC, total cholesterol; LDL-C, low-density lipoprotein cholesterol.

**Table 2:** Univariate associations between bilirubin and demographic, biochemical parameters and non-invasive liver fibrosis indices in participants with HBV infection.

Parameters	TBil		IBil		DBil	
	rho	*p*-Value	rho	*p*-Value	rho	*p*-Value
Age (years)	0.11	<0.001	0.06	0.01	0.18	<0.001
BMI (kg/m^2^)	−0.03	0.18	−0.01	0.66	−0.06	0.01
WHR	0.08	0.00	0.08	0.00	0.05	0.03
ALT (U/L)	0.01	0.54	0.03	0.24	−0.01	0.66
AST (U/L)	0.05	0.03	0.02	0.36	0.10	<0.001
Hemoglobin (g/L)	0.23	<0.001	0.21	<0.001	0.19	<0.001
Platelets count (10^9^/L)	−0.25	<0.001	−0.20	<0.001	−0.27	<0.001
TG (mg/dL)	−0.11	<0.001	−0.04	0.05	−0.19	<0.001
TC (mg/dL)	−0.15	<0.001	−0.06	0.01	−0.27	<0.001
LDL-C (mg/dL)	−0.07	0.00	0.00	0.97	−0.18	<0.001
APRI	0.18	<0.001	0.13	<0.001	0.22	<0.001
FIB4	0.24	<0.001	0.16	<0.001	0.32	<0.001

TBil, total bilirubin; IBil, indirect bilirubin; DBil, direct bilirubin; BMI, body mass index; WHR, waist-to-hip ratios; ALT, alanine transaminase; AST, aspartate transaminase; TG, triglycerides; TC, total cholesterol; LDL-C, low-density lipoprotein cholesterol; APRI, aspartate transaminase to platelet ratio index; FIB-4, Fibrosis 4 score; rho: Spearman’s rank correlation coefficient.

**Table 3:** Odds ratios and 95% CIs for serum TBil levels and the presence of elevated levels of APRI or FIB-4 in HBsAg (+) individuals.

Fibrotic Indices	Q1	Q2	Q3	Q4	*p* for Trend
	(0.00~0.70)	(0.70~0.90)	(0.90~1.16)	(1.16~)	
	*n* = 482	*n* = 482	*n* = 484	*n* = 490	
APRI ≥ 0.5 ^a^	1.00 [reference]	1.31 (0.94, 1.82)	1.95 (1.42, 2.68)	2.55 (1.88, 3.47)	<0.001
Multivariate model 1 ^b^	1.00 [reference]	1.25 (0.89, 1.74)	1.83 (1.33, 2.52)	2.26 (1.65, 3.11)	
Multivariate model 2 ^c^	1.00 [reference]	1.27 (0.90, 1.78)	1.78 (1.29, 2.47)	2.23 (1.61, 3.09)	
Multivariate model 3 ^d^	1.00 [reference]	1.26 (0.90, 1.78)	1.78 (1.28, 2.47)	2.22 (1.60, 3.08)	
FIB4 ≥ 1.45 ^a^	1.00 [reference]	1.56 (1.17, 2.07)	1.85 (1.38, 2.47)	2.70 (1.98, 3.69)	<0.001
Multivariate model 1 ^b^	1.00 [reference]	1.54 (1.13, 2.09)	1.75 (1.27, 2.40)	2.33 (1.66, 3.27)	
Multivariate model 2 ^c^	1.00 [reference]	1.59 (1.16, 2.19)	1.72 (1.24, 2.39)	2.32 (1.63, 3.31)	
Multivariate model 3 ^d^	1.00 [reference]	1.57 (1.14, 2.17)	1.64 (1.18, 2.28)	2.24 (1.57, 3.21)	

TBil, total bilirubin; APRI, aspartate transaminase to platelet ratio index; FIB-4, Fibrosis 4 score. ^a^ Without adjustment; ^b^ Adjusted for age (continuous), sex (male, female); ^c^ Adjusted for the same set of variables in model 1 plus BMI (continuous), WHR (continuous), smoking (never smoking, quit smoking, current smoking), drinking (never drinking, quit drinking, current drinking), education (≤6/7–9/10–12/≥13), marriage status (yes/no) and physical activity (yes/no); ^d^ Adjusted for the same set of variables in model 2 plus the components of the medical history as dichotomized variables.

**Table 4:** Areas under the receiver operating characteristic curve (AUROC) for elevated fibrotic indices for the TBil, IBil and DBil in HBsAg (+) participants.

Fibrotic Indices	TBil	IBil	DBil
APRI ≥ 0.5	0.61 (0.58, 0.64)	0.59 (0.56, 0.62)	0.63 (0.60, 0.66)
FIB4 ≥ 1.45	0.61 (0.58, 0.64)	0.57 (0.54, 0.60)	0.65 (0.62, 0.68)

Tbil, total bilirubin; IBil, indirect bilirubin; DBil, direct bilirubin; APRI, aspartate transaminase to platelet ratio index; FIB-4, Fibrosis 4 score; HBsAg, hepatitis B surface antigen.

## Supplementary Materials

Supplementary materials can be found at www.mdpi.com/1422-0067/17/12/2057/s1.

## Abbreviations

AUROC	areas under the receiver operating characteristic curve
ALT	alanine transaminase
APRI	aspartate transaminase to platelet ratio index
AST	aspartate transaminase
BMI	body mass index
CHC	chronic hepatitis C
CIs	confidence intervals
CHD	coronary heart disease
DBil	direct bilirubin
FIB-4	fibrosis 4 score
HBV	hepatitis B virus
HBsAg	hepatitis B surface antigen
HCV	hepatitis C virus
HCC	hepatocellular carcinoma
IBil	indirect bilirubin
LDL-C	low-density lipoprotein cholesterol
ORs	odds ratios
SD	standard deviation
TBil	total bilirubin
TC	total cholesterol
TG	triglycerides
WHR	waist-to-hip ratios
