# Supplementary material for: The Relationship between Serum Bilirubin and Elevated Fibrotic Indices among HBV Carriers: A Cross-Sectional Study of a Chinese Population
Source: Int J Mol Sci. 2016 Dec 9;17(12):2057. doi: 10.3390/ijms17122057 (PMC5187857; doi:10.3390/ijms17122057)
Supplement: Supplementary file 1 [file ijms-17-02057-s001.pdf]

Supplementary Materials: Relationship between Serum Bilirubin and Elevated Fibrotic Indices among HBV Carriers: A Cross-Sectional Study among Chinese Population

Min Du, Shanshan Zhang, Lin Xiao, Yanyan Xu, Peiyi Liu, Yuhan Tang, Sheng Wei, Mingyou Xing, Xiaoping Miao and Ping Yao

Table S1. Odds ratios and 95% CIs for serum IBil levels and the presence of elevated levels of APRI or FIB-4 in HBsAg(+) individuals.

Fibrotic Indices	Q1 (0.00~0.46) n = 481	Q2 (0.46~0.61) n = 472	Q3 (0.61~0.78) n = 500	Q4 (0.78~) n = 485	p for Trend
APRI ≥ 0.5 [a]	1.00 [reference]	1.34 (0.97, 1.85)	1.59 (1.16, 2.18)	2.33 (1.72, 3.16)	<0.001
Multivariate model 1 [b]	1.00 [reference]	1.30 (0.94, 1.80)	1.55 (1.13, 2.12)	2.13 (1.57, 2.91)	
Multivariate model 2 [c]	1.00 [reference]	1.32 (0.95, 1.84)	1.53 (1.11, 2.12)	2.18 (1.59, 2.99)	
Multivariate model 3 [d]	1.00 [reference]	1.30 (0.93, 1.82)	1.52 (1.10, 2.11)	2.16 (1.57, 2.98)	
FIB4 ≥ 1.45 [a]	1.00 [reference]	1.24 (0.93, 1.65)	1.38 (1.03, 1.83)	1.98 (1.45, 2.69)	<0.001
Multivariate model 1 [b]	1.00 [reference]	1.25 (0.92, 1.71)	1.38 (1.00, 1.88)	1.80 (1.29, 2.52)	
Multivariate model 2 [c]	1.00 [reference]	1.29 (0.94, 1.79)	1.45 (1.04, 2.00)	1.83 (1.29, 2.58)	
Multivariate model 3 [d]	1.00 [reference]	1.24 (0.89, 1.72)	1.41 (1.01, 1.96)	1.75 (1.24, 2.49)	

IBil, indirect bilirubin; APRI, aspartate transaminase to platelet ratio index; FIB-4, Fibrosis 4 score. [a] Without adjustment; [b] Adjusted for age (continuous), sex (male, female); [c] Adjusted for the same set of variables in model 1 plus BMI (continuous), WHR (continuous), smoking (never smoking, quit smoking, current smoking), drinking (never drinking, quit drinking, current drinking), education (≤6/7–9/10–12/≥13), marriage status (yes/no) and physical activity (yes/no); [d] Adjusted for the same set of variables in model 2 plus the components of the medical history as dichotomized variables.

Table S2. Odds ratios and 95% CIs for serum DBil levels and the presence of elevated levels of APRI and FIB-4 in HBsAg(+) individuals.

Fibrotic Indices	Q1 (0.00~0.22) n = 462	Q2 (0.22~0.29) n = 489	Q3 (0.29~0.38) n = 500	Q4 (0.38~) n = 487	p for Trend
APRI ≥ 0.5 [a]	1.00 [reference]	1.39 (0.99, 1.95)	1.62 (1.17, 2.25)	3.22 (2.36, 4.41)	<0.001
Multivariate model 1 [b]	1.00 [reference]	1.33 (0.95, 1.86)	1.50 (1.08, 2.10)	2.84 (2.05, 3.94)	
Multivariate model 2 [c]	1.00 [reference]	1.29 (0.91, 1.82)	1.41 (1.00, 1.98)	2.64 (1.90, 3.69)	
Multivariate model 3 [d]	1.00 [reference]	1.30 (0.92, 1.84)	1.42 (1.01, 2.00)	2.64 (1.89, 3.70)	
FIB4 ≥ 1.45 [a]	1.00 [reference]	1.64 (1.25, 2.17)	2.25 (1.69, 3.01)	4.17 (2.98 5.82)	<0.001
Multivariate model 1 [b]	1.00 [reference]	1.56 (1.15, 2.11)	1.91 (1.40, 2.61)	3.38 (2.35, 4.87)	
Multivariate model 2 [c]	1.00 [reference]	1.49 (1.09, 2.03)	1.83 (1.32, 2.54)	3.10 (2.13, 4.51)	
Multivariate model 3 [d]	1.00 [reference]	1.42 (1.04, 1.95)	1.73 (1.24 2.40)	3.07 (2.10, 4.50)	

DBil, direct bilirubin; APRI, aspartate transaminase to platelet ratio index; FIB-4, Fibrosis 4 score. [a] Without adjustment; [b] Adjusted for age (continuous), sex (male, female); [c] Adjusted for the same set of variables in model 1 plus BMI (continuous), WHR (continuous), smoking (never smoking, quit smoking, current smoking), drinking (never drinking, quit drinking, current drinking), education (≤6/7–9/10–12/≥13), marriage status (yes/no) and physical activity (yes/no); [d] Adjusted for the same set of variables in model 2 plus the components of the medical history as dichotomized variables.
